# Psychosocial Well-Being Among Adult Residents of Flood-Prone Communities in Trinidad: Associated Factors and Predictors in a Cross-Sectional Study

**DOI:** 10.7759/cureus.80045

**Published:** 2025-03-04

**Authors:** Mandreker Bahall, George Legall, Rahvisha Rampersad, Nishana Chatoorie, Arianne Youksee, Erin Wallace, Rae-Anna Choutie, Yelena Singh

**Affiliations:** 1 Caribbean Center for Health Systems Research and Development, University of the West Indies, St. Augustine Campus, Marabella, TTO; 2 Faculty of Medical Sciences, University of the West Indies, Mt. Hope, TTO

**Keywords:** depression, flooding, psychological status, ptsd, stress

## Abstract

Background

Globally, flooding is one of the severe consequences of climate change, which is exacerbated by urbanization and inadequate infrastructure. In Trinidad and Tobago, floods affect a sizable proportion of the population and pose a major public health hazard.

Aim

This study assessed the psychosocial impact of flooding on adult residents in flood-prone areas of Trinidad and Tobago, focusing on the prevalence of mental health conditions such as depression, anxiety, stress, and post-traumatic stress disorder (PTSD), along with identifying associated factors and predictors of psychological distress.

Methods

Convenience sampling was used to collect data from 215 adult household residents in four selected flood-prone areas in Trinidad and Tobago over 12 weeks, from January 2024 to March 2024. The inclusion criteria were being at least 18 years of age, having resided in their area for at least two consecutive years prior to the start date of the study, and having experienced at least one severe flood in the two years prior to the start date. The data collection instrument was a questionnaire, which was chosen to ensure broad accessibility and ease of data collection in the selected flood-prone areas. The variables measured included selected sociodemographic characteristics, household medical history, and flood characteristics. The 36 items also included the following: (1) the Patient Health Questionnaire-2, (2) the Generalized Anxiety Disorder 2-item, (3) the Perceived Stress Scale 4, and (4) the Posttraumatic Stress Disorder Checklist. In this study, the nature of flooding was categorized ordinally as none, mild, moderate, and severe, in keeping with the flood classifications in Queensland in 2020. Descriptive and inferential statistical methods were used for data analysis.

Results

Of the 215 residents, 154 (71.6%) met the eligibility criteria and were invited to complete the online questionnaire. Participants were primarily female (n = 162, or 75.3%), predominantly Indo-Trinbagonians (n = 136, or 63.3%), and had a university or college education (n = 129, or 60%). Among the victims, 127 (82.5%) needed to be evacuated. More than four-fifths (82.5%) of those who experienced flooding were classified as having moderate-to-severe stress, with "Age group" being a key predictor; slightly over half (53.2%) experienced no depression, and 56.5% experienced no anxiety. The reported psychological symptoms included mild depression (34.4%), mild anxiety (36.4%), and mild PTSD (38.3%). The differences between or among mean psychological symptom scores were not significant for any sociodemographic variable. However, "Need to evacuate," "Age group," "Flood duration," and "Flood severity" were associated with at least one of anxiety, PTSD, and stress (p < 0.05). Ordinal logistic regression showed that (1) "Need to evacuate" was a predictor of anxiety, (2) "Flood severity" and "Need to evacuate" were predictors of PTSD, and (3) "Age group" was the only predictor of stress. These findings suggest that evacuation may serve as a critical stressor leading to higher anxiety and PTSD symptoms.

Conclusion

Flood victims experienced significant psychosocial problems with stress, followed by PTSD as the most common disorder. Implementing targeted psychological support and community preparedness programs could mitigate the psychosocial effects of flooding. Follow-up studies with broader populations are needed to help assist in further subgroup analysis.

## Introduction

Globally, flooding is one of the severe consequences of climate change. However, according to Fazir Khan, “other factors such as development, drainage, legislative, administrative, and infrastructure issues” also significantly contribute to flooding in Trinidad and Tobago, which has seen a pronounced elevation in the frequency and magnitude of flooding in recent years [1]. This increase in the incidence and intensification of flooding in recent years has led to “several areas in Trinidad that have not had a flood in years experiencing massive flooding [2],” with hundreds of thousands of citizens being impacted on a yearly basis [3]. As flooding events increase in both frequency and intensity, so too do the mental health consequences among affected individuals. Indeed, in addition to financial, property, and personal losses caused by flooding, there are significant mental health consequences, including stress, anxiety, post-traumatic stress disorder (PTSD), depression, and substance abuse [4]. Victims often report feelings of “grief, panic, loss, fear, and sadness,” alongside difficulties such as insomnia, anger, irritability, and guilt [5]. However, despite the growing frequency of flooding, there have been no studies in the past decade examining the psychosocial impact of flooding in the English-speaking Caribbean region [6]. In Trinidad and Tobago, while the Ministry of Environment continues to document the effect of annual flooding in rural and urban areas, little has been done through quantitative research to assess its impact on households in general and individuals within households in particular. Furthermore, while one study explored the socio-economic impact, the study did not explore the mental health consequences of flood victims [7].

The study aimed to (1) describe the psychosocial impact of flooding on adult residents in flood-prone areas, (2) identify household-level factors that are associated with or predict the psychosocial impact, and (3) identify predictors of impact levels on residents in four flood-prone regions in Trinidad and Tobago. This study specifically sought to determine the prevalence of mental disorders, such as depression, anxiety, stress, and PTSD, and to identify associated factors and predictors. Furthermore, while case-control studies often rely on control regions unaffected by floods, the authors intentionally opted for a cross-sectional study design, focusing on flood-prone areas to better capture the real-time effects on residents. This study lays the groundwork for a follow-up study, which will further explore the impacts in non-flood-prone areas for comparison.

A modified version of this study was presented as a poster at the National Health Research Conference in Trinidad and Tobago on November 22, 2024.

## Materials and methods

The target population consisted of adults from four flood-prone regions of Trinidad - North-East, North-West, Central, and South regions - chosen for their varying geographical and demographic characteristics. Severe flooding was defined according to the Queensland 2020 flood classification, which categorizes flood events based on their severity, impact on infrastructure, and community response [8]. The study focused on households that experienced at least one severe flood in the two years prior to the study, as severe flooding is likely to result in significant psychosocial impacts. The inclusion criteria were as follows: (1) residing in one of the four regions where data were to be collected for at least two contiguous years before the start date of the study; (2) being at least 18 years of age and knowledgeable of the experiences of the household; (3) households that experienced at least one severe flood in the two years prior to the start date.

Convenience sampling was used to recruit the participants through online surveys distributed via social media platforms and local networks to ensure a diverse sample representative of the target regions. The questionnaire was pre-tested in a small sample prior to the study to ensure the clarity and reliability of the items. The estimated time for completing the questionnaire was based on pilot testing, with an average completion time of 15 minutes. Consenting participants completed the self-completion design questionnaire consisting of 36 items: socio-demographics (10), medical history (5), flooding characteristics (11), psychosocial screening (6), and brief self-reported psychological assessment (4), using the following instruments: Patient Health Questionnaire-2 (PHQ-2) to assess depression, Generalized Anxiety Disorder 2-item (GAD-2) to assess anxiety, PTSD Checklist to assess PTSD, and Perceived Stress Scale 4 to assess stress. The psychosocial screening and psychological assessments were self-reported measures designed to capture the mental health impact of flooding, with established reliability and validity. The PHQ-2 was chosen to assess depression symptoms due to its established validity in community-based studies, while the GAD-2 and PTSD Checklist were selected for their efficacy in measuring anxiety and PTSD, respectively. Details of the data collection instruments can be found in the Appendices (Table 9). Prior to analysis, scores for depression, anxiety, PTSD, and stress were categorized using an ordinal scale based on established cutoff points, as detailed in Table 1.

**Table 1:** Score ranges and levels PTSD, post-traumatic stress disorder

	Score range	Levels by range of total scores	
		None - Mild	Moderate - Severe
Depression	0 - 6	0 - 2	3 - 6
Anxiety	0 - 6	0 - 2	3 - 6
PTSD	0 - 24	0 - 6	7 - 24
Stress	0 - 16	0 - 5	6 - 16

Statistical analysis

Descriptive and inferential data analyses were conducted. The former included frequency and percentage distribution tables, means and standard deviations of scores, sample percentages, and bar charts. The latter included Pearson’s product-moment correlations with corresponding p-values, analysis of variance (ANOVA) to compare mean scores, chi-square tests of association between qualitative variables, and ordinal logistic regression to identify predictors of depression, anxiety, PTSD, and stress. Pearson’s product-moment correlations were used to determine whether or not the functional relationship between a given pair of continuous variables was linear. Chi-square tests of association were used to test for independence, or lack of association, between two categorical values. Ordinal logistic regression was used as the primary method for identifying predictors due to the ordinal nature of the psychosocial outcomes, which were categorized into severity levels. In addition, multiple comparisons (pairwise) were used only when the F-test showed that the null hypothesis of equality was not rejected. Tukey's method was used because it has been shown to be conservative. All hypotheses were tested at the 5% level of significance (p ≤ 0.05).

Ethical considerations

The participants were informed of the nature and purpose of the study. Informed consent was obtained from all participants before data collection, and confidentiality was strictly maintained throughout the study. Participants were assured that their responses would remain anonymous and would be used only for research purposes. Ethical approval for this study was obtained from the University of the West Indies Ethics Board and the Ministry of Health Ethical Board of Trinidad and Tobago (approval number: CREC-SA.2419/11/2023).

## Results

By the end of the data collection period, 215 participants were screened. Of these, 154 (71.6%) met the eligibility criteria and were invited to complete the online questionnaire. Of this group, the majority (n = 115, or 74.7%) were female, close to two-fifths (n = 63, or 40.9%) were 18-25 years of age, 93 (60.4%) were of Indo-Trinbagonian descent, a little under 40% (n = 61, or 39.6%) of participants were from the North-Central region, 77.9% (n = 120) of participants had lived in the same area for more than 15 years, and the majority (n = 102, or 66.2%) had completed Secondary School, while the remaining 33.8% (n = 52) had only completed Primary School (Table 2).

**Table 2:** Distribution of sociodemographic variables of flood victims

Variable		Number, or N = 154	%
Sex			
Male	39		25.3
Female	115		74.7
Age group			
18-25	63		40.9
26-35	36		23.4
36-45	33		21.4
45 and over	22		14.5
Ethnicity			
Afro-Trinbagonian	25		16.2
Indo-Trinbagonian	93		60.4
Mixed	35		22.7
Other	1		0.6
Area of residence			
North-Central	61		39.6
South	28		18.2
East	46		29.9
North-West	13		8.4
Other (unspecified)	6		3.9
Length of residence			
Less than 5 years	13		8.4
5-10	11		7.1
11-15	10		6.5
>15	120		77.9

Age was the only sociodemographic variable significantly associated with whether participants had experienced flooding in the two years prior to the start of the study (chi-square = 9.621, df = 3, p = 0.022).

The majority of participants described the flooding they experienced as moderate (n = 66, or 42.9%), lasting a few hours (n = 90, or 58.4%), and 80% (n = 124) reported needing to evacuate, while a larger percentage (n = 127, or 82.5%) stated that the flood necessitated rebuilding or relocation (Table 3).

**Table 3:** Distribution of flood-related variables

Variable	Number, or N = 154	%
Flood severity		
Mild	56	36.4
Moderate	66	42.9
Severe	32	20.8
Flood duration		
Less than 1 hour	11	7.1
A few hours	90	58.4
Days	44	28.6
Weeks	1	0.6
No response	8	5.2
Need to evacuate		
No	30	19.5
Yes	124	80.5
Need to rebuild/relocate		
No	27	17.5
Yes	127	82.5

Depression, anxiety, stress, and PTSD levels

The reliability/internal consistency (Cronbach’s alpha) of each of the four instruments, ranging from "Good" to "Excellent," is presented in Table 4.

**Table 4:** Instrument reliability/internal consistency PTSD, post-traumatic stress disorder

Component	Number of items	Cronbach alpha	Reliability
Depression	2	0.868	Good
Anxiety	2	0.910	Excellent
Stress	6	0.937	Excellent
PTSD	4	0.884	Good

**Figure 1:**
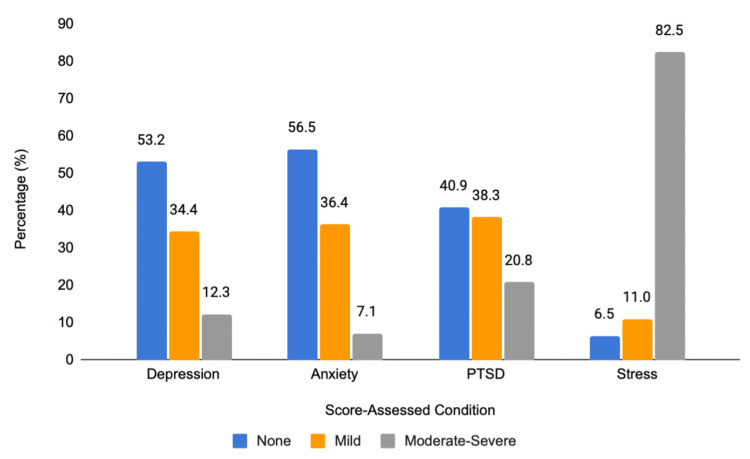
Percentage distribution of levels of depression, anxiety, PTSD, and stress (n = 154) Note: Moderate and severe categories were combined into moderate-severe because, for each condition, only approximately 5% were classified as severe. PTSD, post-traumatic stress disorder

The percentage distributions of depression, anxiety, and stress levels are shown in Figure 1.

Over four-fifths (82.5%) of those who experienced flooding developed moderate-to-severe stress, followed by mild to moderate-to-severe PTSD (59.1%), and mild and moderate-to-severe depression and anxiety (46.7% and 43.5%, respectively).

Summary statistics: attribute scores

The selected summary statistics for the total anxiety, depression, PTSD, and stress scores are presented in Table 5. Subsequent inferential methods included ANOVA, chi-square test of association, and ordinal logistic regression.

**Table 5:** Scores summary statistics (anxiety, depression, PTSD, stress) n, number; SD, standard deviation; PTSD, post-traumatic stress disorder

Attribute	n	Mean	SD	Minimum	Median	Maximum
Anxiety	154	1.08	1.475	0.0	0.0	6.0
Depression	154	1.01	1.512	0.0	0.0	6.0
PTSD	154	3.73	5.060	0.0	2.0	24.0
Stress	154	7.17	2.309	0.0	8.0	16.0

Table 6 presents the p-values obtained using ANOVA to compare mean scores (anxiety, depression, PTSD, and stress) among the categories of sociodemographic variables. No significant differences were observed among the mean scores for any of the four conditions (p > 0.05).

**Table 6:** p-values from ANOVA methods to compare mean scores ANOVA, analysis of variance; PTSD, post-traumatic stress disorder

	Condition: p-values			
	Anxiety	Depression	PTSD	Stress
Sex	0.615	0.480	0.599	0.755
Age group	0.333	0.572	0.396	0.456
Ethnicity	0.430	0.725	0.490	0.739
Area of residence	0.8512	0.726	0.441	0.812
Length of residence	0.269	0.352	0.339	0.146
Level of education	0.430	0.931	0.298	0.342

Correlations and associations

Table 7 presents the bivariate correlation coefficients (Pearson product-moment) among the total depression, PTSD, stress, and anxiety scores. The only significant associations were (i) between PTSD and stress (p = 0.050), and (ii) between PTSD and anxiety (p ≤ 0.001).

**Table 7:** Bivariate correlation coefficients **p-value ≤0.05 signifies a non-zero correlation. PTSD, post-traumatic stress disorder

	Depression	PTSD	Stress	Anxiety
Depression	1	-0.018 (0.795)	0.038 (0.584)	-0.035 (0.608)
PTSD	-0.018 (0.795)	1	0.134 (0.050**)	0.616 (≤0.001**)
Stress	0.038 (0.584)	0.134 (0.050**)	1	0.104 (0.129)
Anxiety	-0.035 (0.608)	0.616 (≤0.001**)	0.104 (0.129)	1

The association between age and stress (chi-square = 15.959, df = 6, p = 0.014) was the only association between psychological characteristics and demographic variables.

Other associations were: (1) flood duration and PTSD level: chi-square = 15.521, df = 2, p < 0.001; (2) flood severity and PTSD level: chi-square = 35.768, df = 4, p < 0.001; (3) need to evacuate and PTSD level: chi-square = 21.129, df = 2, p < 0.001; and (4) need to evacuate and anxiety level: chi-square = 26.680, df = 2, p < 0.001.

Logistic regression: predictors

"Flood severity" and "Need to evacuate" were predictors of PTSD, "Need to evacuate" was a predictor of anxiety, and "Age group" was a predictor of stress (Table 8).

**Table 8:** Predictors of PTSD, anxiety, and stress *Significant at the 5% level; empty cells in this table are necessarily present because they represent the reference category for each variable. OR, odds ratio; CI, confidence interval; PTSD, post-traumatic stress disorder

	Variable	OR	p-value	95% CI for OR	
				Lower	Upper
PTSD	Flood duration				
	Days	0.962	0.768	0.746	1.242
	Weeks	1	-	-	-
	Flood severity				
	Mild	0.465	0.001*	0.326	0.662
	Moderate	0.579	0.001*	0.417	0.804
	Severe	1	-	-	-
	Need to evacuate				
	No	1.479	0.009*	1.013	0.982
	Yes	1	-	-	-
Anxiety	Need to evacuate				
	No	1.924	0.001*	1.532	2.415
	Yes	1	-	-	-
Stress	Age group				
	18-25	1.257	0.046*	1.004	1.575
	26-35	1.462	0.003*	1.331	1.885
	36-45	1.292	0.258	0.991	1.684
	Over 45	1	-	-	-

## Discussion

This study revealed that a significant proportion (n = 154, or 71.6%) of the people in flood-prone areas experienced flooding problems. Age was the only sociodemographic variable significantly associated with whether participants had experienced flooding in the two years before the start of the study (chi-square = 15.959; df = 6, p = 0.014). Although the association between age and flooding was statistically significant, this unexpected result may have been influenced by the study’s non-randomized and online sampling bias, which could have overrepresented certain age groups who are more likely to respond to online surveys. In our study, the association between gender and flooding level was not established, and logistic regression was not used. The majority of participants described the flooding they experienced as moderate (n = 66, or 42.9%) and lasting a few hours (n = 90, or 58.4%). Eighty percent (n = 124) reported needing to evacuate, whereas a larger percentage (n = 127, or 82.5%) stated that the flood necessitated rebuilding or relocation. The psychosocial impact of flooding is indeed a major cause for concern, as was reported in a meta-analysis conducted in the UK by Cruz et al. in 2020, which stated that "water depth in the house was associated with increased risk of psychological distress" [9]. Furthermore, a 2022 UK study by Twiddy et al. found that, compared to those not personally affected by flooding, those affected by flooding had worsened mental health outcomes, with an increased likelihood of experiencing "high levels of anxiety when storms are forecast" [10].

Our study revealed that stress was the most common psychological factor affecting nearly all flood victims (93.5%). This may stem from the uncertainty and apprehension flood victims face, as well as the financial burden arising from recovering after a flooding event, as noted by Mulchandani et al. in a 2019 study that performed a secondary analysis of cross-sectional survey data from the English National Study of Flooding and Health, which stated that flood victims with insurance issues experienced severe stress [11]. The average stress levels in Trinidad and Tobago have not been reported, but are presumably quite high.

PTSD in our study revealed mild to moderate to severe PTSD (59.1%), with moderate to severe accounting for 20.8% of the population among flood victims. Similar findings were reported by Mao et al. in a 2022 study on the prevalence of PTSD one year after a flood in Fort McMurray, Northern Alberta, Canada, which found a 39.6% prevalence of PTSD [12]. Additionally, a paper published in 2013 among persons aged 60 or older found that affected individuals "reported significantly higher PTSD symptoms, with about one in six reporting PTSD symptoms that might require clinical attention," compared with persons who were not affected and experienced "a greater increase in anxiety post-flood" [13]. Furthermore, a 2015 meta-analysis of flood-related PTSD in English and Chinese literature published between 1980 and 2013 by Chen and Liu found a significant difference in "the incidence of PTSD among floods of different trauma intensities," with severe flood trauma resulting in a higher "incidence of PTSD compared with that of mild flood trauma" [14].

Our study reveals that mild and "moderate to severe" depression affected 46.7% of flood victims, which is similar to other studies, such as a UK study conducted by Munro et al. in 2017, which reported that people who had no warning of flooding and displacement "were significantly more likely to report symptoms of depression" [15]. Furthermore, a study by Akpinar-Elci et al. in Guyana found that flood victims had "a slight non-significant increase risk of depression" [16]. Depression levels in other subpopulations in Trinidad had a similar prevalence, with a clinical depression prevalence of 40% "among hospitalized patients with cardiac disease" [17].

Anxiety levels (mild and moderate to severe) were reported at 43.5%, which is relatively high compared to general anxiety levels reported in other studies, such as the Munro et al. study, which found that only 22% of participants who were flooded but not displaced had probable anxiety, whereas only 30% of participants who were flooded and displaced had probable anxiety [15]. This may have resulted from a lack of coping mechanisms, such as family, support groups, and governmental intervention.

Similar to our study, a 2019 study conducted in Cumbria, England, found that participants affected by flooding had poorer mental health outcomes than those who were unaffected [18]. However, among the four mental disorders studied, no interaction was observed among these conditions, although other studies have reported otherwise. For instance, a 2019 study published by Mulchandani et al., which found that flood victims with severe stress due to insurance issues had an increased incidence of probable depression, PTSD, and anxiety compared with those with mild or no stress [11].

Limitations of methodology

This study had some limitations with respect to the study design, which affected our ability to conduct the research effectively. The following were the main limitations: (1) sampling was not randomized because it was not possible to construct a sampling frame of eligible households or to use area sampling, which can lead to significant bias and limit generalizability; (2) the use of an online platform, while convenient, restricted the sample to more technologically literate individuals, which may have led to the exclusion of older populations, those unwilling to use devices for surveys, or those in areas with limited internet access. This limits the generalizability of the findings to the broader flood-affected population; (3) in addition to the inadequate sample size, the study may have lacked sufficient power to detect subtler relationships between socio-economic factors and mental health outcomes. Future studies with larger sample sizes that are randomized could provide more robust insights; (4) given the reliance on self-reported data, the findings may have been affected by recall bias or social desirability bias. To minimize these biases, future studies could offer more detailed instructions and potentially utilize mixed methods to verify subjective responses; (5) subgroup analysis, such as sex, housing, and income, could not be tested because of the small sample size. Despite these limitations, this study reported important findings. In light of our limitations, future studies should be done involving face-to-face interviews with a bigger sample and a wider cross-section in areas of flooding to help improve reliability and assist in further subgroup analysis.

Recommendations

Immediate psychological support should include trauma-informed counseling, mental health hotlines, and community-based peer support networks. Long-term mental health services should also be established, focusing on addressing the cumulative impact of repeated flooding events. Additionally, enhanced preparedness and targeted mental health services are essential to mitigate these impacts. Community-based disaster preparedness programs, along with government initiatives to train local mental health professionals, are essential for improving resilience in flood-prone populations.

## Conclusions

Most victims experienced moderate-to-severe flooding. Flood victims face significant psychosocial challenges. Stress affects nearly the entire population, while PTSD, anxiety, and depression affect approximately half. The severity and duration of flooding and the need to evacuate are associated with psychological factors; however, the severity of flooding and the need to evacuate were the only predictors of the selected psychological factors. The findings suggest that the severity and duration of flooding, along with the need to evacuate, are significant determinants of psychosocial distress. Future interventions should prioritize these factors to alleviate the mental health burden on flood victims. Given the significant psychosocial impact of flooding, future studies should explore the specific mechanisms through which evacuation and flood severity influence mental health, as this could guide the development of more targeted interventions in flood-prone areas.

## Appendices

**Table 9:** Questionnaire Note: *Participant’s response to an open-ended question.

Number	Question	Possible answer
SECTION A: Sociodemographic (Please choose the most appropriate response for each question)		
1.	How old are you?	*
2.	What is your sex?	Male
		Female
3.	Please indicate your ethnicity	Afro-Trinidadian
		Indo-Trinidadian
		Mixed
		Chinese
		Other: *
4.	Where do you reside?	Arima
		Couva/Tabaquite/Talparo
		Diego Martin
		Penal/Debe
		Princes Town
		Sangre Grande
		San Juan/Laventille
		San Fernando
		Siparia
		Mayaro/Rio Claro
		Chaguanas
		Point Fortin
		Port of Spain
		Tunapuna/Piarco/Caroni
		Other: *
5.	How long have you been living in this area?	Less than 5 years
		5 to 10 years
		11 to 15 years
		More than 15 years
6.	What is your highest educational level achieved?	Primary
		Secondary
		University/College
		None
7.	What is your current employment status?	Employed
		Unemployed
		Retired
		Student
8.	Who do you live with? (You may select more than one option)	Alone
		Spouse
		Children
		Friend
		Parent(s)
		Other: *
9.	Do you have the following? (You may select more than one option)	Family Support
		Social Support
		Financial Support
10.	What is your average income per month? (Can answer if you feel comfortable)	Less than $3,999
		$4,000-$6,999
		$7,000-$9,999
		More than $10,000
SECTION B: Flooding Characteristics (Please choose the most appropriate response for each question)		
11.	Have you ever experienced flooding events in your residing area? If "no", proceed to Section C	Yes
		No
12.	When was the last time you experienced a flood?	Less than 3 months
		4-6 months
		7-12 months
		More than 12 months
13.	How often has your area experienced flooding events in the past 2 years?	1-2 times
		3-5 times
		More than 5 times
		I am unsure
14.	How would you describe the severity of flooding in your area? (Please refer to the characteristics to determine your answer)	Mild (Causes inconvenience; Low lying areas next to watercourses are inundated. Minor roads may be closed and low-level bridges submerged; In urban areas inundation may affect some backyards and buildings below the floor level as well as bicycle and pedestrian paths; In rural areas removal of stock and equipment may be required)
		Moderate (In addition to the Minor flood effects, the area of inundation is more substantial; Main traffic routes may be affected; Some buildings may experience water above the floor level; Evacuation of flood affected areas may be required. In rural areas removal of stock is required)
		Severe (In addition to the Moderate flood effects, extensive rural areas and/or urban areas are inundated; Many buildings may be affected above the floor level; Properties and towns are likely to be isolated and major rail and traffic routes closed; Evacuation of flood affected areas may be required; Utility services may be impacted)
15.	How long does a flooding event usually last in your region?	Few minutes
		Hours
		Days
		Weeks
		I am unsure
16.	Are there specific triggers that typically lead to flooding in your region? (You may select more than one option)	Poor drainage
		Live near rivers
		Heavy rainfall
		Storm surges
		I am unsure
		Other: *
17.	What is the maximum water level height during a flood?	<0.5 (1.7ft)
		0.6 m - 1.0 m (1.8 ft - 3.3 ft)
		1.1 m - 2.0 m (3.4 ft - 6.6 ft)
		2.1 m - 3.0 m (6.7 ft - 9.9 ft)
		>3.0 m (>10 ft)
		I am unsure
18.	Did you ever have to evacuate due to flooding?	Yes
		No
19.	Have you ever had to rebuild or move out from your current house due to severe flooding?	Yes
		No
20.	On a scale of 1-10, how prepared are you for a flooding event? (1 being the least prepared and 10 being the most prepared)	*
21.	How many days after a flooding event have you been unable to attend work or school?	0 days
		1-2 days
		3-5 days
		1 week
		More than 1 week
SECTION C: Medical History For Questions 22-24: Please indicate if you have suffered from any of the following conditions, and if they worsened during or after a flooding event		
22. Chronic Diseases	I) Heart Disease	No
		Yes
		If ‘yes’, has it worsened during or after a flooding event?
	ii) High Blood Pressure or Hypertension	No
		Yes
		If ‘yes’, has it worsened during or after a flooding event?
	iii) High Blood Cholesterol	No
		Yes
		If ‘yes’, has it worsened during or after a flooding event?
	iv) Congestive or Chronic Heart Failure	No
		Yes
		If ‘yes’, has it worsened during or after a flooding event?
	v) Stroke (with continued weakness from the event)	No
		Yes
		If ‘yes’, has it worsened during or after a flooding event?
	vi) Diabetes (sugar in the blood)	No
		Yes
		If ‘yes’, has it worsened during or after a flooding event?
	vii) Chronic Bronchitis or Emphysema	No
		Yes
		If ‘yes’, has it worsened during or after a flooding event?
	viii) Asthma	No
		Yes
		If ‘yes’, has it worsened during or after a flooding event?
	ix) Kidney Disease (other than an infection or a stone)	No
		Yes
		If ‘yes’, has it worsened during or after a flooding event?
	x) Sinus Problems	No
		Yes
		If ‘yes’, has it worsened during or after a flooding event?
	xi) Digestive Issues	No
		Yes
		If ‘yes’, has it worsened during or after a flooding event?
23. Mental Health Disorder	i) Depression	No
		Yes
		If ‘yes’, has it worsened during or after a flooding event?
	ii) Anxiety	No
		Yes
		If ‘yes’, has it worsened during or after a flooding event?
	iii) Bipolar Disorder	No
		Yes
		If ‘yes’, has it worsened during or after a flooding event?
	iv) Schizophrenia	No
		Yes
		If ‘yes’, has it worsened during or after a flooding event?
24. Autoimmune Disease	i) Rheumatoid Arthritis	No
		Yes
		If ‘yes’, has it worsened during or after a flooding event?
	ii) Lupus	No
		Yes
		If ‘yes’, has it worsened during or after a flooding event?
	iii) Multiple Sclerosis	No
		Yes
		If ‘yes’, has it worsened during or after a flooding event?
25.	Is it difficult to access medical treatment during or after a flooding event? (medical treatment - hospital, doctor, pharmacy, dialysis treatment, etc) If ‘no’ or ‘maybe’, proceed to Section D	Yes
		No
		Maybe
26.	Why was it difficult to access medical treatment during or after a flooding event?	*
SECTION D: Psychosocial screening		
27. Assessing Depression: PHQ-2 Scale (For each of the following questions, please select the response that best describes how you felt DURING or TWO WEEKS AFTER a flooding event.)	i) Little interest or pleasure in doing things	Not At All
		Several Days
		More than Half the Days
		Nearly Every Day
	ii) Feeling down, depressed, or hopeless	Not At All
		Several Days
		More than Half the Days
		Nearly Every Day
28. Assessing Anxiety: GAD-2 Scale (For each of the following questions, please select how often you have been bothered by the following problems DURING or TWO WEEKS AFTER a FLOODING event)	i) Feeling nervous, anxious or on edge	Not At All
		Several Days
		More than Half the Days
		Nearly Every Day
	ii) Not being able to stop or control worrying	Not At All
		Several Days
		More than Half the Days
		Nearly Every Day
29. Assessing Post Traumatic Stress Disorder: Abbreviated PCL-C Scale (For each of the following 6 questions, please select the response that best describes how you felt DURING or TWO WEEKS AFTER a flooding event)	i) Repeated, disturbing memories, thoughts, or images of a stressful experience from the past?	Not At All
		A Little Bit
		Moderately
		Quite a Bit
		Extremely
	ii) Feeling very upset when something reminded you of a stressful experience from the past?	Not At All
		A Little Bit
		Moderately
		Quite a Bit
		Extremely
	iii) Avoided activities or situations because they reminded you of a stressful experience from the past?	Not At All
		A Little Bit
		Moderately
		Quite a Bit
		Extremely
	iv) Feeling irritable or having angry outbursts?	Not At All
		A Little Bit
		Moderately
		Quite a Bit
		Extremely
	v) Difficulty concentrating?	Not At All
		A Little Bit
		Moderately
		Quite a Bit
		Extremely
	vi) Feeling jumpy or easily startled?	Not At All
		A Little Bit
		Moderately
		Quite a Bit
		Extremely
30. Assessing Stress: Abbreviated PSS-10 (For each of the following 4 questions, please select the response that best describes how you felt DURING or ONE MONTH AFTER a flooding event)	i) How often have you felt that you were unable to control the important things in your life?	Never
		Almost Never
		Sometimes
		Fairly Often
		Very Often
	ii) How often have you felt confident about your ability to handle your personal problems?	Never
		Almost Never
		Sometimes
		Fairly Often
		Very Often
	iii) How often have you felt that things were going your way?	Never
		Almost Never
		Sometimes
		Fairly Often
		Very Often
	iv) How often have you felt difficulties were piling up so high that you could not overcome them?	Never
		Almost Never
		Sometimes
		Fairly Often
		Very Often
31.	Have you experienced the loss of a family member or loved one due to flooding events in your community? If ‘no’, move to Question 33.	Yes
		No
32.	How did this affect your family and community?	*
33.	What portion of your monthly income is allocated to flooding preparation and management?	None at All
		A Small Amount
		Approximately Half
		More than Half
		A Significant Portion
34.	Have you experienced the loss of property due to flooding events in your community? If ‘No’, move to the end of questionnaire.	Yes
		No
35.	Please select the extent of the property loss and the specific items or assets that were affected (You may select more than one option).	House
		Vehicle
		Furniture
		Kitchen Appliances (Fridge, Stove, etc)
		Jewelry
		Important documents
		Books
		Electronics (Phones, Tablets, Laptops, Television, etc)
		Family Photos/Heirlooms
		General Appliances (Washing Machines)
		Other: *
36.	How did this property loss impact you and your family?	*
End of questionnaire, thank you for your assistance and time! We appreciate your feedback.		
